# Validation of a new sampler for radon gas measurements in surface water

**DOI:** 10.1016/j.mex.2024.102815

**Published:** 2024-06-21

**Authors:** Gustavo S. Luís, Alcides J.S.C. Pereira, José Carvalho, Luís F. Neves

**Affiliations:** a Univ Coimbra, CITEUC - Centre for Earth and Space Research, Department of Earth Sciences, Portugal; b Univ Coimbra, Department of Earth Sciences, Portugal

**Keywords:** Radon, Rn-222, Liquid scintillation counting, Surface water, Sampling, New sampler for radon gas measurements in surface water

## Abstract

Radon gas (Rn-222) in water poses health risks due to radiation exposure, yet it's also an important tracer for studying natural systems. Sampling procedures for Rn-222 analysis are very sensitive to potential losses of the gas to the atmosphere. It requires a well-defined and properly validated protocol to ensure accuracy and reliability. A novel sampler was developed to collect surface water from a distance (e.g. from bridges), addressing logistic challenges posed by topography. The sampler, manually operated, ensures precise depth-specific sampling throughout the water column. A three-stage validation process (technical performance test, uncertainty estimations and preliminary test) was followed to validate the protocol.

• The comparison of the technical procedure for analysis and measurement through Liquid Scintillation Counting is statistically robust (one-way ANOVA p-value = 0.96).
• For the protocol proposed for Rn-222 determination, the estimated sampling and measurement uncertainties (*k* = 2) are respectively 5% and 15%. These are compatible with the literature and the laboratory's precision.
• Preliminary tests, with meaningful patterns identified and possibly related to the river's hydrodynamics, revealed a very reliable protocol, even in low Rn-222 concentrations.

Therefore, the sampler has demonstrated a good analytical reproducibility and was considered validated for Rn-222 determination in surface waters.

## Graphical abstract

**Figure:**
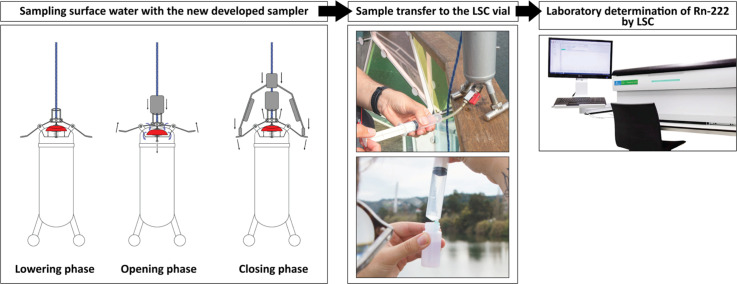

Specifications table

**Table:** 

Subject area:	Environmental Sciences
More specific subject area:	Natural radioactivity
Name of your method:	New sampler for radon gas measurements in surface water
Name and reference of original method:	Not applicable.
Resource availability:	New sampler developed is a prototype and it isn't commercially available. The sampling methodology is applicable to any measurement technique for Rn-222 in water therefore no specific resources are needed.

## Background

Rn-222 is a natural radioisotope (half-life of 3.82 days) that decays from Ra-226. Its concentration in drinking water is a health concern due to the increase of exposure to radiation of a human body through water ingestion [[1], [19]]. On the other hand, it is also a valuable tracer for the study of natural systems, like the groundwater and surface water interactions [2,3].

Rn-222 is a noble gas that can easily desorb from water to the atmosphere, thus water sampling and sample handling can be an important source of error and uncertainty to the result to be reported (ISO 13164–4:2023). For reliable and comparable results is therefore fundamental a validated and consistent protocol articulated with good sampling practices [[4], [20]].

An extensive work by Jobbágy et al. [5] addressed some of these principal sources of error. Another important step not covered there or in ISO 13164–4:2023 [16] is the water sampling when no direct access to the sampling location is available (e.g., wells, boreholes, piezometers, or surface waters without easy access). In these cases, a sampler must be used (e.g., pumps, sampling bailers, or bottle shaped samplers) and another possible source of error is introduced.

Topography with steep-sided V-shaped valley may difficult the direct and safe access to the river. For these situations a new sampler, inspired in the well-know Niskin and Van Dorn samplers, was developed at the Laboratory of Natural Radioactivity (LRN) of the Department of Earth Sciences of the University of Coimbra, especially designed to be operated at the distance from bridges above the sampling location. The main goal of this sampler is to collect representative and reproducible surface water for Rn-222 analysis at different depths. This is especially important to characterize all the water column with significant depth, from the water surface to the riverbed.

In this work, the novel sampler is firstly introduced and presented. The validation of the protocol and the sampler is carried out to ensure reliable results for future works.

## Method details

### The sampling protocol

#### New surface water sampler and its operational protocol

**Fig. 1:**
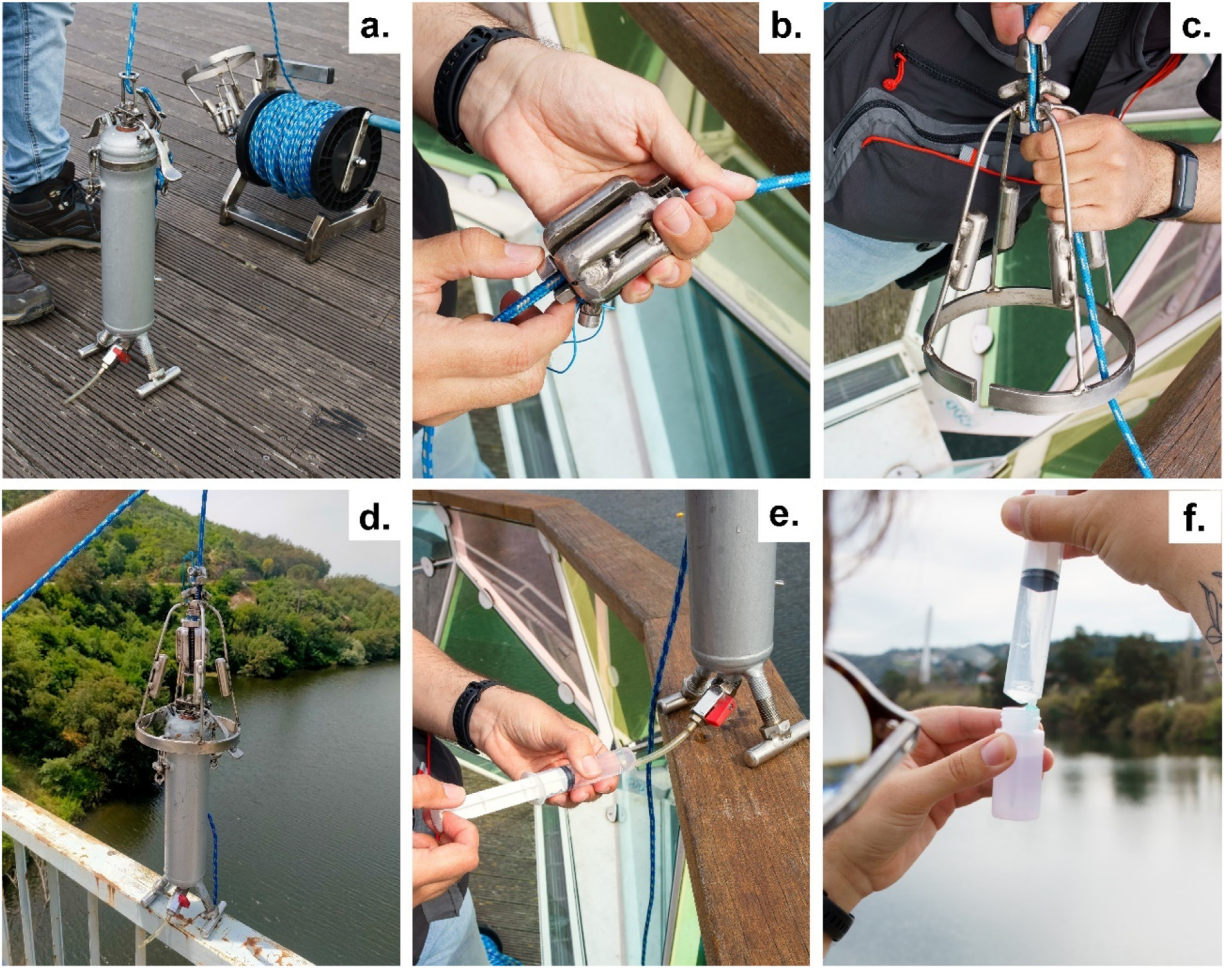
Sampling of surface water for Rn-222 analysis: a. sampler and its accessories; b. weight messenger used to open the sampler at the desired depth; c. weight messenger used to close the sampler at the desired depth; d. sampler and its mechanisms after the sampling; e. collecting water from the bottom of the sampler with a gas-tight syringe coupled to the sampler; f. injection of the sampled water inside a pre-prepared vial, under an immiscible scintillation cocktail.

The developed sampler is a metallic bottle-shape with a volume of approximately 1 L (Fig. 1.a.). The sampler is free of electronics. The operability relies on a central system of levers and spring that act over the closing lid of the sampler. This is activated by two different weight messengers that are responsible for triggering the opening (Fig. 1.b.) and the closing (Fig. 1.c.) stage. These are dropped down a resistant rope (climbing grade) attached to the sampler (Fig. 1.d.). A simple and cheap diode light (used for fishing) can be attached at the lid level. This emits flashing light when in contact with water. Then the operator can control the water depth of the sampler. The metallic body and components make it a rugged equipment to handle all types of weather conditions and its implications to the flow regime of the surface waters. In the field we've found some adverse weather conditions that even the metallic body weight wasn't enough to vertically lower the sampler. In these scenarios extra weights can be attached to both sampler's feet.

**Fig. 2:**
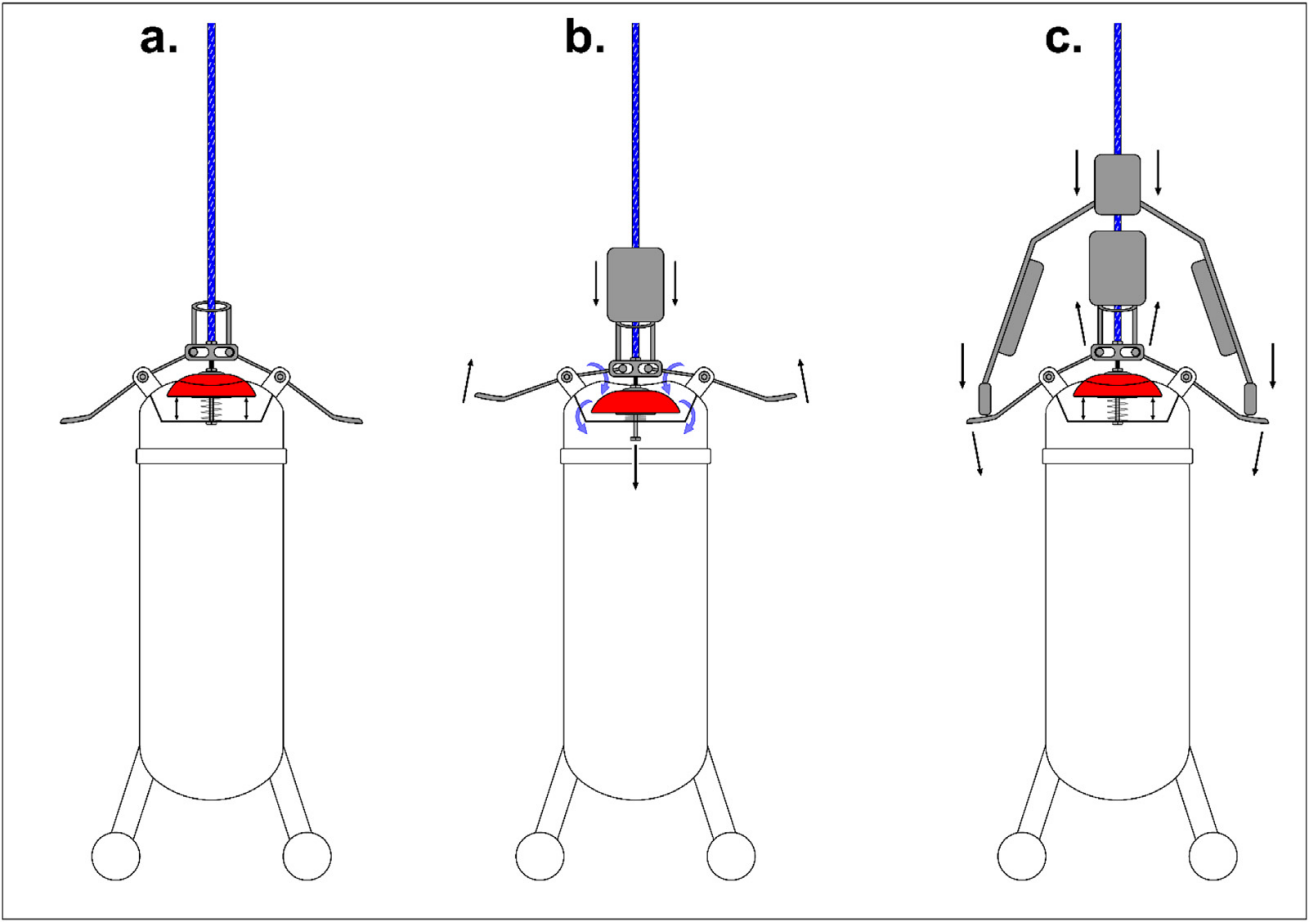
Scheme of the central mechanism of the sampler and the weights messengers to open and close the lid, according to a. lowering phase; b. opening phase; and c. closing phase.

The in-depth surface water sampling procedure is divided into three main stages (Fig. 2):

a. Lowering phase – The sampler is lowered attached to a metric rope from a higher location where the operator is (e.g., a bridge). When it touches the surface of the water the operator feels the tension lowering. When the lid touches the water, the operator can visualize it or the diode light. From this point the sampler can be lowered in the water column to the desired depth completely watertight.
b. Opening phase – At the desired depth the operator drops the first weight through the rope, which will trigger the opening mechanism.
c. After a calibrated time (approximately one and half minutes) the sampler is filled. The second weight is dropped and it triggers the lever that is responsible for closing the sampler. The sampler is then lifted back to the operator.

The three main characteristics of this sampler are: i) its simplicity in its own construction and to operate; ii) its capability for opening and close the sampler at the desired depth, without the need for free flow of water during the lowering phase of the sampler; and iii) it is already equipped with a valve and tygon tube for the coupling of a gas-tight syringe for Rn-222 sampling. The relative compact size of the rope reel, already prepared for the transport of the messengers (Fig. 2.a.), and the sampler, as also its simple mechanisms, are especially important for field operation in such a way that a single operator can handle the entire procedure.

#### In-situ sample preparation for liquid scintillation counting

Samples for Rn-222 analysis through Liquid Scintillation Counting (LSC) are prepared in the field following the methodology described in Belloni et al. [6]. Water is taken from the sampler or from the desired water source (e.g., a fountain spout) using a gas-tight syringe accoupled to the sampler by a tygon tube (Fig. 1.e.). 10 mL of the water sampled are injected inside a LSC Teflon vial under an immiscible scintillation cocktail (Betaplate Scint), previously prepared (Fig. 1.f.). This procedure should be smooth and as fast as possible to prevent radon loss to the atmosphere. After sealing the vial, it was vigorously shaken to fasten the radon extraction by the scintillation cocktail. The vial was kept at rest for at least 3 h before the measurement for Rn-222 to reach secular equilibrium with its short-lived progeny.

## Determination of RN-222 activity concentration by liquid scintilation counting

Radon activity concentrations (RC) were determined in the Laboratory of Natural Radioactivity at the University of Coimbra (Portugal), which is under the scope of ISO/IEC 17025:2017 [17], for the determination of RC following the ISO 13164–4:2023 [16]. Samples were analyzed using a liquid scintillation counter Quantulus GCT 6220 from Perkin Elmer, after reaching the secular equilibrium. No alpha-beta discrimination was used to reach a higher efficiency. That could be a trade-off with higher background, but in this LSC apparatus it is only possible to enable the equipment's Guard Compensation Technology (GCT) in cpm counting mode (no discriminator) for background reduction. The protocol also prevents the measurement from another source of uncertainty (estimation of the optimal discriminator) and error (spillover of alfa and beta particles).

RC at the time of sampling was determined according to equation Eq. 1:

(1) $$RC=\frac{CR_{S}-CR_{B}}{E\cdot m\cdot e^{-\lambda t}}\cdot \rho \;$$

where RC is the Rn-222 concentration (Bq *L*^−1^), CR_S_ is the count rate of the sample (cps), CR_B_ is the count rate of the blank sample (cps), E is the detection efficiency (Bq cps^−1^), m is the mass of the tested sample (g), ρ is the water density (assumed to be 1000 g.dm^−3^), λ is Rn-222 decay constant and t is the time elapsed between sampling and counting. The detection limit (DL) of the technique for the defined measuring protocol is estimated to be 56 mBq *L*^−1^, following the ISO Standard [16]. For statistical analysis the results below DL were substituted by 0.65×DL following [18].

## Validation stages

**Fig. 3:**
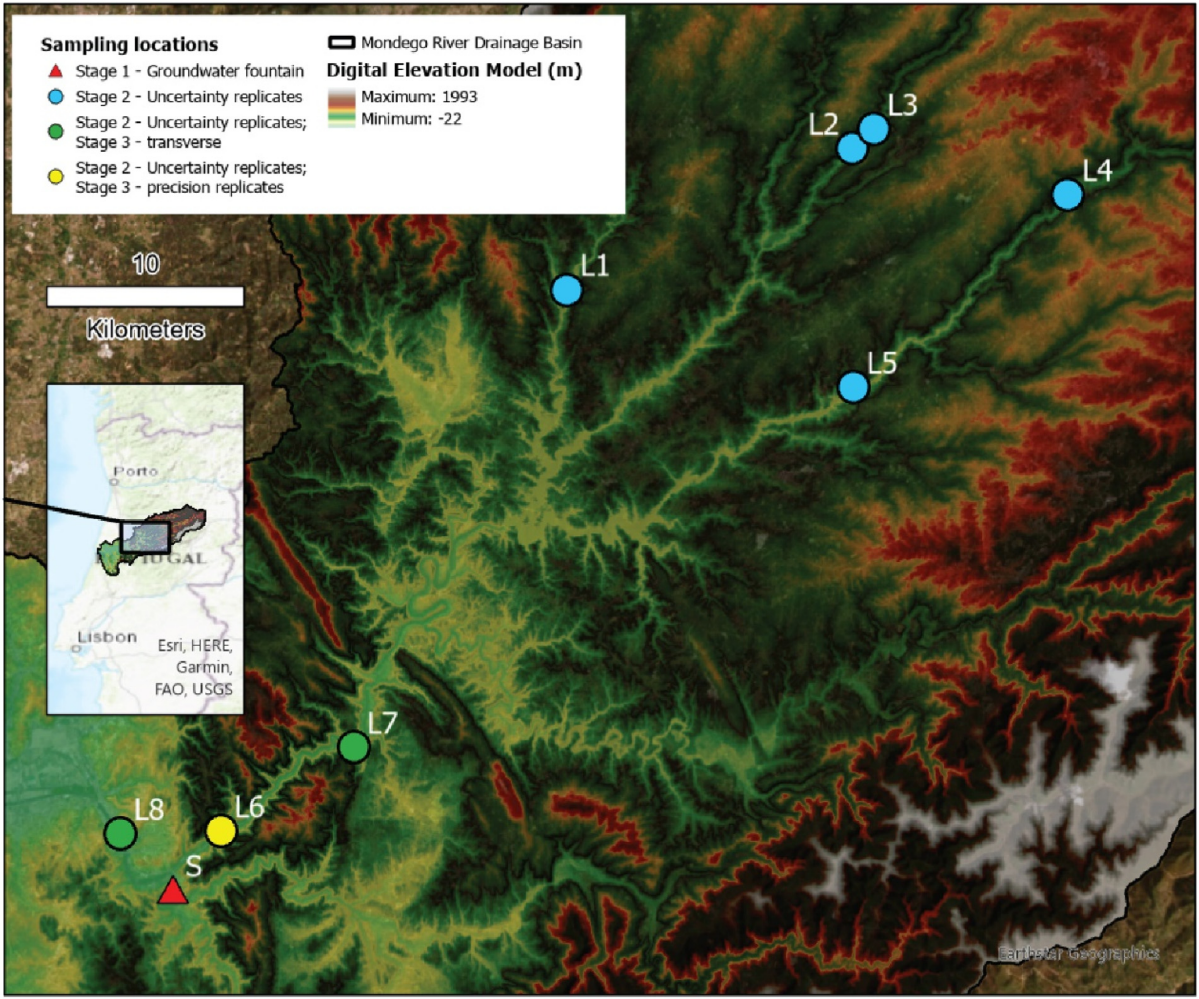
Sampling locations of spring water (S) of stage 1 and surface waters (L) of stage 2 and 3. L numbers are in accordance with Table 2, for stage 2. Locations with transverse surveys and precision evaluation (stage 3) were also used for uncertainty estimates (stage 2).

Aiming to validate all the sampling protocol proposed, field tests were carried out in the Mondego River drainage basin (Fig. 3), in the center of Portugal. The sampling protocol, as already presented, is divided into two separated procedures that could be a source of uncertainty: i) the instrumental procedure of the sampler in depth and independent of the operator; ii) the technical procedure for the preparation of the sample for LSC. Then the validation of the methodology was divided into three stages: i) evaluation of the technical intervention in the preparation of the sample *in-situ* (without the presented sampler); ii) uncertainty estimation of the sampling protocol with the newly developed sampler through a replicate design; and iii) final test of the sampler and its protocol in a real situation.

### Stage 1 – performance comparison of the technician intervention in-field sample preparation

In this first stage it was intended to evaluate the technical performance of the sampling technician without the use of the developed sampler for future comparison with the sampler's protocol uncertainties. The comparison of the sampling performance (stage 1) of two technicians for the analysis of radon in water was performed in a groundwater public fountain in a sedimentary outcrop of the upper Triassic (Conraria Formation). Replicate samples were sampled randomly intercalated by both technicians. During the sampling interval (15 min), technician 1 collected 10 samples and technician 2 collected 13 samples.

Precision was evaluated through the relative standard deviation (RSD), calculated according to Eq. 1a:

(1a) $$RSD=\frac{\sqrt{\frac{\sum {\left(X-\bar{X}\right)}^{2}}{n-1}}}{\bar{X}}$$

where X are the sample values, $\bar{X}$ is the sample mean and n is the number of observations.

### Stage 2 – uncertainty estimation of the sampling protocol

**Fig. 4:**
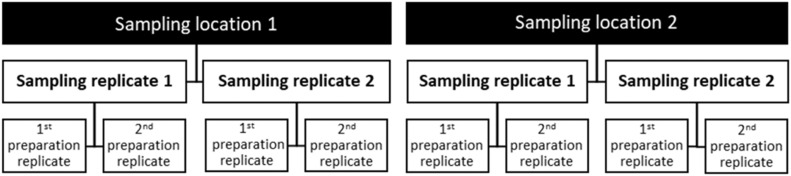
Replicate design with two split levels used to estimate the sampling, analytical and measurement uncertainties.

A large-scale uncertainty evaluation was conducted at the Mondego river basin. At 8 different sampling locations at different lithological outcrops (sedimentary, metamorphic and igneous, with expected different RC) it was followed a replicate design [7] with two split levels (Fig. 4). With this design it is possible to estimate the sampling, the analytical and the combined uncertainties. At each sampling location, the sampler was lowered two times to the same depth and collected one water sample each time (sampling replicates 1 and 2). For each time the sampler was lowered, two samples were prepared from the sampler (preparation replicates 1 and 2).

Following the statistical model presented in Eq. 2 [7] a two-way analysis of variance (ANOVA) with replicates estimates the analytical ($s_{a}^{2}$) and the sampling ($s_{s}^{2}$) variances.

(2) $$x_{meas}=X_{true}+\varepsilon_{sampling}+\varepsilon_{analytical}$$

Where $x_{meas}$ is the measured value, $X_{true}$ is the true value and ε are the random analytical and sampling errors. Sampling ($u_{s}$) and analytical ($u_{a}$) relative uncertainties are estimated according to Eq. 3 and 4:

(3) $$u_{a}=\frac{\sqrt{s_{a}^{2}}}{mean}$$

(4) $$u_{s}=\frac{\sqrt{s_{s}^{2}}}{mean}$$

The combined standard uncertainty of the measurement ($u_{m}$) is then calculated following Eq. 5:

(5) $$u_{m}=\sqrt{u_{s}^{2}+u_{a}^{2}}$$

For a 95% confidence interval (CI), uncertainties are expanded by a coverage factor k=2 (Eq. 6).

(6) U=2·u

### Stage 3 – preliminary real-life test

In this stage a brief preliminary testing was performed to understand if Rn-222 concentrations in surface water would reflect the expected river hydrodynamics. For that, at two different sections of the river, samples of water were collected with the sampler following a transect to the river flow. At location PI the river channel is linear and location L is a bend of the river. At a third location in-between both sections, three replicates were sampled at the exact same location and depth to evaluate if precision results are compatible with the uncertainties estimated in the previous stage.

## Data analysis

### Method validation

Descriptive statistic and hypothesis testing (Shapiro-Wilk Normality test, F-test for variances and one-way ANOVA) were performed using R Language through Stats package [8]. Estimation of sampling, analytical and measurement uncertainties (stage 2), for the convenience of having a dedicated software, were run in RANOVA3 software [9].

## Results

### Stage 1 – performance comparison of the technician intervention in-field sample preparation

RC results of both technicians are presented in Table 1. Shapiro-Wilk test confirm that should be assumed that both technicians’ results are normally distributed. Significative F-test indicate that variances of both technicians aren't equal, with the RSD being 3% and 8% for technician 1 and 2, respectively. Nevertheless, means should not be considered different.

**Table 1:** Results of Rn-222 concentration of replicates and statistical analysis (descriptive and hypothesis testing statistics) of stage 1 sampling performance comparison of two technicians for Rn-222 analysis. The differences in observations is due to the protocol: both technicians had 15 min for sampling.

*Sample count*	*Technician 1 Rn-222 (Bq L^−1^)*	*Technician 2 Rn-222 (Bq L^−1^)*
*1*	*87*	*80*
*2*	*85*	*82*
*3*	*88*	*92*
*4*	*86*	*91*
*5*	*87*	*91*
*6*	*81*	*74*
*7*	*89*	*85*
*8*	*86*	*81*
*9*	*84*	*84*
*10*	*88*	*80*
*11*		*88*
*12*		*96*
*13*		*95*

*Mean*	*86Bq L^−1^*	*86Bq L^−1^*
*Standard Deviation*	*2Bq L^−1^*	*7Bq L^−1^*
*Variance*	*5Bq L^−1^*	*43Bq L^−1^*
*% Relative Standard deviation (RSD)*	*3*	*8*

*Observations*	*10*	*13*
*Shapiro-Wilk Normality Test*	*W=0.913 p-value = 0.301*	*W=0.954 p-value = 0.664*

*F-Test for variances*	*F=0.109 DF1 = 9; DF2 = 12 p-value = 0.002*	
*One-way ANOVA*	*F=0.003 DF = 1 p-value = 0.958*	

### Stage 2 – uncertainty estimation of the sampling protocol

The RC averages in surface waters of the eight selected locations dispersed from 901 mBq *L*^−1^ to 25 000 mBq *L*^−1^. RC results and two-way ANOVA results are shown in Table 2. The estimated relative uncertainty arising from the sampling procedure of surface water with the newly developed sampler is 4.9% (95% CI). The estimated uncertainty due to the analytical procedure is 14.6% (95% CI). The combined uncertainty of the measurement is 15.4% (95% CI).

**Table 2:** Radon concentration (mBq *L*^−1^) results and ANOVA results of the replicate design with two split levels for the estimation of uncertainty. In the SiPi designation, S refers to the sample replicate and P to the preparation replicate.

	*S1P1 Rn-222 (mBq L^−1^)*	*S1P2 Rn-222 (mBq L^−1^)*	*S2P1 Rn-222 (mBq L^−1^)*	*S2P2 Rn-222 (mBq L^−1^)*
*Location 1*	*1 782*	*1 685*	*1 504*	*1 369*
*Location 2*	*9 157*	*9 979*	*9 393*	*9 037*
*Location 3*	*24 035*	*25 734*	*26 121*	*25 654*
*Location 4*	*1 110*	*1 072*	*647*	*774*
*Location 5*	*2 008*	*1 788*	*1 499*	*1 873*
*Location 6*	*1 080*	*837*	*853*	*993*
*Location 7*	*881*	*1 135*	*830*	*852*
*Location 8*	*919*	*1 384*	*992*	*1 463*

	*Between Target*	*Sampling*	*Analysis*	*Measurement*
*Standard Deviation*	*8.62*	*0.13*	*0.38*	*0.40*
*% of total variance*	*99.78*	*0.02*	*0.20*	*0.22*
*U% (relative uncertainty) (95%)*		*4.9*	*14.6*	*15.4*

### Stage 3 – preliminary real-life test

**Fig. 5:**
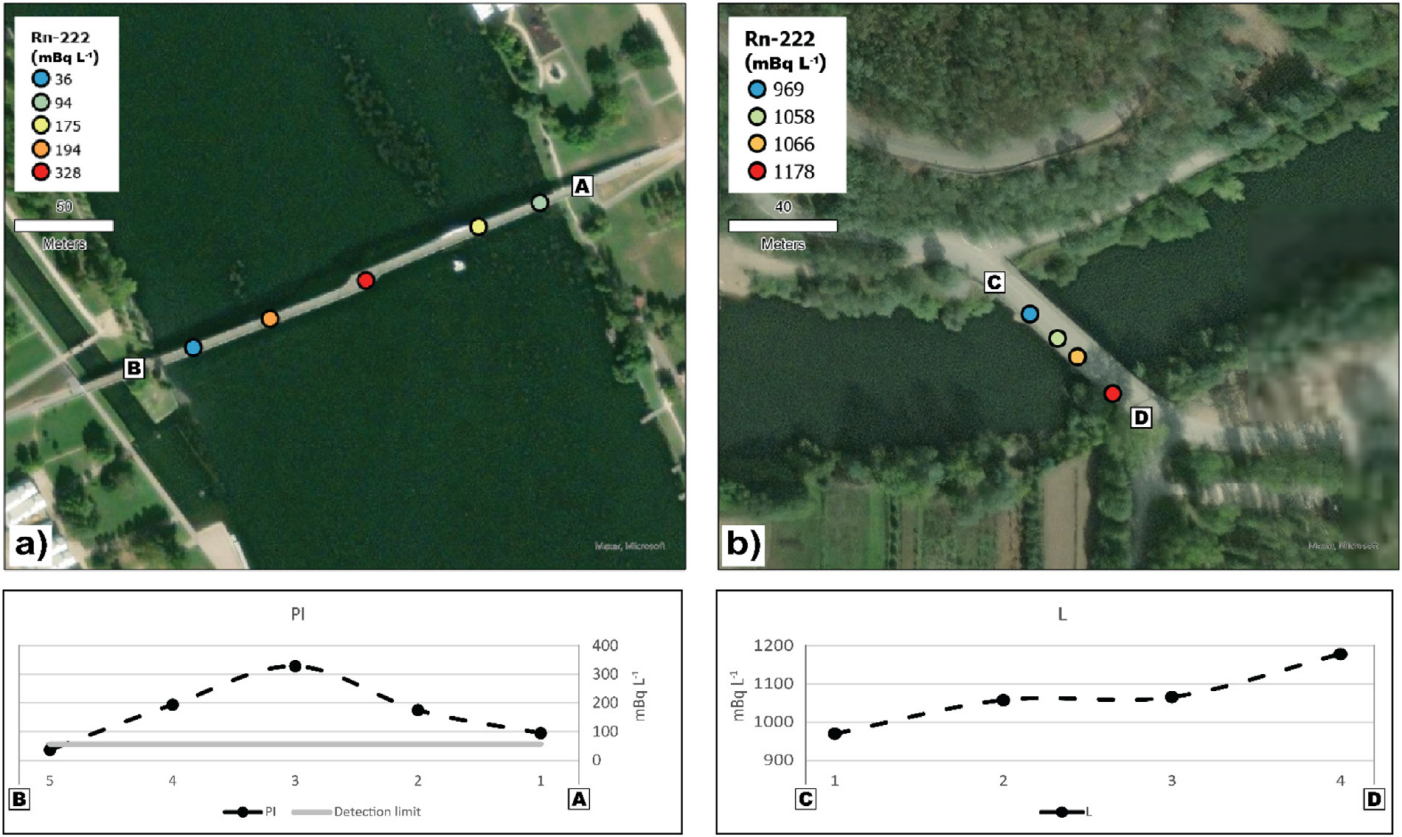
Spatial and graphical projection of Rn-222 concentration in surface waters of two transects surveys (PI and L) to the river. Results smaller than the detection limit (56 mBq *L*^−1^) are represented as 36 mBq *L*^−1^ (0.65 x detection limit).

In the transect at location PI (Fig. 5.a.) RC is lower near both margins. From there, RC increases towards the center of the river and reaching a maximum RC of 328 mBq *L*^−1^. At location L (Fig. 5.b.) RC values increase from the inner margin to the outer margin of the river bend. In both transects the deviations around the mean are similar ($\sigma_{PI}=109$ mBq *L*^−1^; $\sigma_{L}=86$ mBq *L*^−1^), which means that RSD is higher (66%) at location PI and lower RSD (8%) at location L. At location T, the three sampling replicates have shown a RC mean of 1237 mBq *L*^−1^ with a standard deviation of 8 mBq *L*^−1^, which represents an RSD of 1%.

## The validation of the protocol

Precision and accuracy reported in the method's validation by the Laboratory of Natural Radioactivity of the University of Coimbra are, respectively, 14.1% and 4.1%. In the sampling comparison of the first stage, both technicians achieved in-field precisions (3% and 8% RSD) compatible with the laboratory precision. No bias between both technicians was identified, whose achieved the same average RC of 86 Bq *L*^−1^. These precisions are comparable to the combined uncertainties derived from the sampling and the analysis uncertainties presented by Bruggeman et al. [10]. Nevertheless, is important to note that even though the precisions are acceptable, variances are significantly different. This is an indication that sampling should be carried out carefully and consistently.

The eight selected locations in stage 2 show a good spread of RC values in surface waters. These RC are representative of the different scenarios found in the Mondego River drainage basin. For the designed test, sampling uncertainty estimate is representative of all the in-field protocol with the newly developed sampler. It comprises simultaneously the uncertainty arising from i) the sampler filling protocol and ii) the syringe injection of the sample into the vial (comparable to the field test comparison of stage 1). Results have shown that sampling uncertainty ($U_{s}=4.9\%$) with the proposed protocol is also comparable to sampling uncertainties between 3 and 5% reported in Bruggeman et al. [10]. This estimated sampling uncertainty is considerably lower than: i) analysis uncertainty ($U_{a}=14.6\%$), ii) precision estimates of stage 1 (RSD=6−16%, for a 95% CI) and iii) laboratory's reported precision (14.1%). In the other hand, analysis uncertainty ($U_{a}=14.6\%$) is comparable to the last mentioned: ii) precision estimates of stage 1, and iii) precision reported by the laboratory. Therefore, combined uncertainty ($U_{m}=15.4\%$) is not strongly affected by the estimated sampling uncertainty. It is in good agreement with typical 8 – 20% range of combined uncertainties (for a 95% CI) for LSC reported in other recent studies [3,5,[10], [11], [12], [13]].

In the final stage of the validation of the newly developed sampler, transect results are considered meaningful and interpreted as a trace of the river's hydrodynamics. In transect PI, RC variability is not justified by the estimated uncertainty. The increasing RC from the margins to the center of the river is compatible with higher river flow in the middle of the river and lower turbulence, compared to the margins. Even though the variability in transect L can be justified by the uncertainty, the same well-defined pattern related to river's flow is found. In the river's bend, RC increase from the inner margin, with slower and more turbulent flow, to the outer margin with higher water flow. Is not to exclude the possibility of trends of preferable groundwater discharges into the surface water. This is a simplistic interpretation of a preliminary test, whose only purpose is to verify that RC sampled with the newly developed sampler is meaningful.

The final three replicates showed a precision of approximately 1% which is in good agreement with the estimates of the previous stages of this study.

From a more practical perspective is also important to discuss that the robustness of a metallic sampler is important for reliable field sampling. Other types of materials (e.g., acrylic) couldn't handle the river's flow in certain conditions during the rainy season: in deeper water columns the sampler didn't submerge into the water and in shallower waters with turbulent flows the sampler could crash with rocks and break.

## Final remarks

Radon is a valuable tracer to complement hydrogeological studies, especially when considering the interaction of groundwater and surface water. Despite the relative high cost of LSC equipment, the sampling and analysis techniques are relatively simple, making possible to fasten the sampling campaigns. But there are other techniques out there to measure radon in-situ at lower costs [14,15]. Regardless of the technique used, the critical point for reliable results of RC is the sampling procedure due to the natural gaseous state of radon.

The sampler presented here has shown good reproducibility of the results and a low sampling uncertainty of approximately 5%, well below the 15% of the estimated combined uncertainty of the measurement. These results are comparable to the uncertainties presented in other published works. After the three-stage testing the protocol with the newly developed sampler is considered accepted.

## Limitations

Although the good results, the protocol still relies much on the sample transfer technique to the LSC vial. This is to say that sampling should be carried out carefully, fast, but without disregarding the smoothness.

## CRediT authorship contribution statement

**Gustavo S. Luís:** Conceptualization, Investigation, Methodology, Data curation, Formal analysis, Writing – original draft, Visualization. **Alcides J.S.C. Pereira:** Validation, Writing – review & editing, Supervision, Resources. **José Carvalho:** Methodology, Visualization, Writing – review & editing. **Luís F. Neves:** Writing – review & editing, Supervision, Resources.

## Declaration of competing interest

The authors declare that they have no known competing financial interests or personal relationships that could have appeared to influence the work reported in this paper.

## Data Availability

Data will be made available on request.
